# Expression of the Astrocyte Water Channel Aquaporin-4 in the Mouse Brain

**DOI:** 10.1177/1759091415605486

**Published:** 2015-10-15

**Authors:** Jacqueline A. Hubbard, Mike S. Hsu, Marcus M. Seldin, Devin K. Binder

**Affiliations:** 1 Center for Glial-Neuronal Interactions, Division of Biomedical Sciences, University of California, Riverside, CA, USA; 2 Division of Cardiology, University of California, Los Angeles, CA, USA

**Keywords:** astrocyte, aquaporin-4, brain, cerebellum, glia limitans, mouse

## Abstract

Aquaporin-4 (AQP4) is a bidirectional water channel that is found on astrocytes throughout the central nervous system. Expression is particularly high around areas in contact with cerebrospinal fluid, suggesting that AQP4 plays a role in fluid exchange between the cerebrospinal fluid compartments and the brain. Despite its significant role in the brain, the overall spatial and region-specific distribution of AQP4 has yet to be fully characterized. In this study, we used Western blotting and immunohistochemical techniques to characterize AQP4 expression and localization throughout the mouse brain. We observed AQP4 expression throughout the forebrain, subcortical areas, and brainstem. AQP4 protein levels were highest in the cerebellum with lower expression in the cortex and hippocampus. We found that AQP4 immunoreactivity was profuse on glial cells bordering ventricles, blood vessels, and subarachnoid space. Throughout the brain, AQP4 was expressed on astrocytic end-feet surrounding blood vessels but was also heterogeneously expressed in brain tissue parenchyma and neuropil, often with striking laminar specificity. In the cerebellum, we showed that AQP4 colocalized with the proteoglycan brevican, which is synthesized by and expressed on cerebellar astrocytes. Despite the high abundance of AQP4 in the cerebellum, its functional significance has yet to be investigated. Given the known role of AQP4 in synaptic plasticity in the hippocampus, the widespread and region-specific expression pattern of AQP4 suggests involvement not only in fluid balance and ion homeostasis but also local synaptic plasticity and function in distinct brain circuits.

## Introduction

The aquaporins (AQPs) are a family of at least 13 molecular water channels that are expressed throughout various mammalian tissues. A number of these proteins have been found in the brain, including AQP1, AQP3, AQP4, AQP5, AQP6, AQP8, AQP9, and AQP12 (Badaut et al., 2014). Aquaporin-4 (AQP4) is the main water channel in the neuropil of the central nervous system and is highly polarized in expression (Nielsen et al., 1997). It is primarily found on astrocytes, particularly on the astrocytic end-feet surrounding capillaries and the blood brain barrier as well as the glia limitans (Nagelhus et al., 2004; Oshio et al., 2004; Costa et al., 2007). AQP4 is highly abundant at sites of fluid transport, including pial and ependymal surfaces in contact with cerebrospinal fluid (CSF), subarachnoid space, and the ventricular system (Nielsen et al., 1997; Rash et al., 1998). Based on its location and expression, it was hypothesized that AQP4 is involved in bidirectional fluid exchange between both the blood and CSF compartments and the brain (Nagelhus et al., 2004).

Studies using AQP4 knockout mice have helped elucidate the role of AQP4 in brain function. Initially generated in 1997 using targeted gene distribution (Ma et al., 1997), AQP4 knockout mice appear normal in phenotype, growth, tissue morphology, neuromuscular function, blood brain barrier function, baseline intracranial pressure, and intracranial compliance (Ma et al., 1997; Manley et al., 2000; Papadopoulos et al., 2004). However, these mice exhibited decreased astrocyte water permeability (Solenov et al., 2004) and a mild urine concentrating deficit (Ma et al., 1997). The use of these knockout mice have revealed that AQP4 is involved in a variety of brain functions including normal cognitive function (Skucas et al., 2011; Scharfman and Binder, 2013), spatial memory (Zhang et al., 2013), K^+^ buffering (Binder et al., 2006), astrocyte migration (Saadoun et al., 2005), cell adhesion (Hiroaki et al., 2006), and regulation of brain extracellular space (Binder et al., 2004).

Abnormalities in water balance play a crucial role in the pathophysiology of several neurological disorders, including cerebral edema, stroke, and epilepsy. Due to impaired AQP4-dependent water clearance, AQP4 knockout mice had higher intracranial pressure and accelerated neurological deterioration in a model of vasogenic edema compared with wild type mice (Papadopoulos et al., 2004). Interestingly, AQP4-deficient mice have increased survival and reduced swelling in models of cytotoxic (cellular) edema (Manley et al., 2000). In the rodent stroke model of transient occlusion of the middle cerebral artery, AQP4 immunoreactivity was rapidly reduced, primarily in regions with vascular damage (Friedman et al., 2009). However, AQP4 expression changes depend on the model used; for example, in the rodent model of transient focal brain ischemia, peaks of swelling and AQP4 expression, particularly the M1 isoform, were observed at 1- and 48-hr after ischemia (Ribeiro Mde et al., 2006; Hirt et al., 2009). In an animal model of temporal lobe epilepsy, hippocampal AQP4 immunoreactivity was downregulated with partial recovery over time (Lee et al., 2012). In addition, AQP4 knockout mice exhibited prolonged seizure duration and slowed K^+^ kinetics, but increased seizure latency, in response to hippocampal electrical stimulation (Binder et al., 2006). Despite the clear functional significance of AQP4 in both the healthy and diseased brain, AQP4 expression patterns in distinct brain areas have been incompletely defined.

Determining the overall spatial distribution of AQP4 throughout the brain could enhance our understanding of the functional relationship between AQP4 and specific regions in the brain. Thus far, the location and abundance of AQP4 protein in the brain has only been partially described and only in certain brain areas (Nielsen et al., 1997; Rash et al., 1998; Wen et al., 1999; Nagelhus et al., 2004; Oshio et al., 2004; Vitellaro-Zuccarello et al., 2005; Costa et al., 2007; Hsu et al., 2011). In this study, we developed sensitive and specific Western blotting and immunohistochemical techniques to provide a comprehensive description of AQP4 expression and localization throughout the entire mouse brain. We found that highest AQP4 protein levels were found in the cerebellum with significantly lower levels in the cortex, hippocampus, and diencephalon. Throughout the brain, AQP4 does not only display targeted expression on glial end-feet surrounding blood vessels but also marked region-specific parenchymal expression was observed.

## Materials and Methods

### Animals

All experiments were conducted in accordance with the guidelines set forth by the National Institute of Health and were approved by the University of California, Riverside Institutional Animal Care and Use Committee (IACUC). Animals were housed under controlled conditions (12-hr light/12-hr dark) and had access to food and water *ad libitum*. Six-week-old wild-type or AQP4 knockout mice on a CD1 background were used for all experiments.

### Western Blot

CD1 mice (*n* = 5) were deeply anesthetized with an intraperitoneal injection of sodium pentobarbital (200 mg/kg) and were transcardially perfused with ice-cold phosphate-buffered saline (PBS) containing protease inhibitors (Roche). Brains were quickly removed from the skull and the cerebral cortex, diencephalon, hippocampus, cerebellum, and brainstem were rapidly microdissected according to the mouse brain atlas of Paxinos and Watson (Paxinos and Franklin, 2001). Tissue was mechanically homogenized in ice-cold radioimmunoprecipitation assay buffer (150 mM NaCl, 1% NP-40, 0.5 sodium deoxycholate, 0.1% SDS, 50 mM Tris, pH 7.5) containing protease inhibitor cocktail (Roche) using a glass dounce tissue grinder (Wheaton). Lysates were centrifuged at 10,000 g for 5 min and the supernatant extracted. Protein concentrations were determined using Bio-Rad BSA detection system and Bio-Tek plate reader. Briefly, 15 µg of protein were electrophoresed through a 12% SDS-PAGE gel with 0.2% SDS with 8 M urea. Samples were then transferred to a nitrocellulose membrane and incubated overnight in a 5% milk in tris-buffered saline with Tween (TBST) blocking solution with rabbit anti-AQP4 (1:1,000; EMD Millipore) and mouse anti-β-actin (1:5,000, Calbiochem). Specificity of our anti-AQP4 antibody was determined previously with the use of AQP4 knockout mice (Binder et al., 2006). The next day, membranes were washed and incubated for 2 hr at room temperature with peroxidase-conjugated goat anti-rabbit and goat anti-mouse secondary antibodies. After several washes, membranes were visualized with ECL chemiluminescence (Pierce) and captured on Hyblot film (Denville). Band intensities were determined using densitometry (ImageJ), and AQP4 levels were normalized to β-actin. Statistical analysis was performed with GraphPad Prism 5 using a one-way ANOVA with post hoc Tukey pairs of columns comparison test. Statistical significance was determined by a *p* value < .05, .01, or .001.

### Immunohistochemistry

Mice were euthanized with 200 mg/kg sodium pentobarbital and transcardially perfused with ice-cold PBS followed by 4% paraformaldehyde. Brains were then post-fixed overnight in 4% paraformaldehyde followed by cryoprotection in 30% sucrose in PBS, both at 4℃. Tissue was frozen in dry ice-cooled isopentane and stored at −80℃ until sectioning. Tissue was cut into either coronal (*n* = 3 mice) or sagittal (*n* = 3 wild type mice and *n* = 3 AQP4 knockout mice) 50 µm thick sections using a cryostat (Leica CM1950). Two sections from every animal of each brain region examined were used, for a total of *n* = 6 per experiment. In addition, both hemispheres were imaged in coronal slices. Sections were quenched in 3% peroxidase for 1 hr, blocked with 5% normal goat serum in PBS for 1 hr, and incubated with rabbit anti-AQP4 (1:200; EMD Millipore) in 0.3% Triton-X-100 overnight at 4℃. The next day, sections were washed and incubated with HRP-conjugated goat anti-rabbit and tyramide from a TSA kit (Molecular Probes/Invitrogen). Slices were washed and mounted on frosted slides (Fisher) with Vectashield (Vector laboratories).

Double immunofluorescence labeling of AQP4 with the cerebellar astrocyte marker brevican was carried out using mouse anti-brevican (1:200, BD Transduction Laboratories) overnight concurrently with rabbit anti-AQP4. Following tyramide development the next day, sections were washed and incubated with Alexa Fluor 594-conjugated anti-mouse IgG (1:100, Invitrogen) for 2 hr at room temperature. Sections were then washed and counterstained with fluorescent Nissl dye for 10 min. After a final wash, sections were mounted with Vectashield.

### Confocal Microscopy

Fluorescent images were obtained using a fluorescent microscope (BX-51, Olympus) with the 10× objective. Confocal microscope images of various brain regions were generated using either the 63× objective and the Zeiss LSM 510 Meta or the 2× objective on the Olympus BX61. Image processing was done using the LSM 510 imaging software (Zeiss) or Slidebook 4.2. Merged images were created using Photoshop CS4 with the photomerge reposition feature.

## Results

### Differential Expression of AQP4 in the Mouse Brain

**Figure 1.**
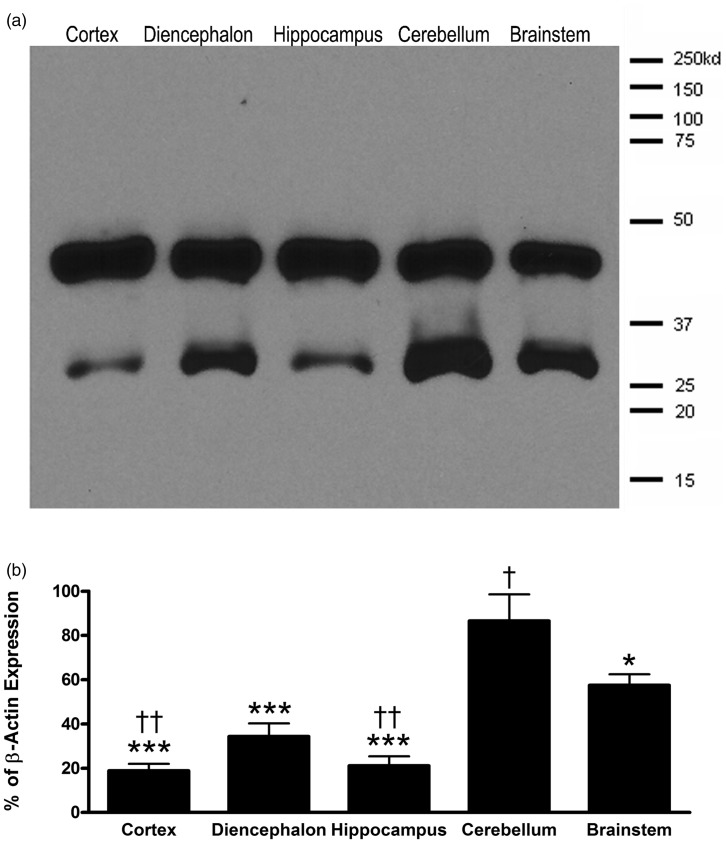
Western blot analysis of various brain regions. (a) Representative blot of AQP4 monomers (∼32–34 Kd, lower band) and β-actin (∼43–45 Kd, upper band) protein in the cortex, diencephalon, hippocampus, cerebellum, and brainstem of the mouse brain. (b) AQP4 band intensities were scanned, quantified, and normalized to the corresponding β-actin band intensities within each brain region (*n* = 3). *indicates *p* < .05 and ***indicates *p* < .001 when compared with the cerebellum. †indicates *p* < .05 and †† indicates *p* < .01 relative to the brainstem.

Western blot analysis revealed an approximately 32 to 34 kDa monomeric band representing AQP4 in the brain. To determine the relative expression levels of AQP4 throughout the brain, protein was isolated from the cortex, diencephalon, hippocampus, cerebellum, and brainstem, separated by SDS-PAGE and probed for AQP4 (Figure 1(a)). Highest expression of AQP4 was found in the cerebellum with a significantly lower amount of AQP4 protein in the hippocampus, diencephalons, and cortex (Figure 1(b)). One-way ANOVA demonstrated a significantly difference between the amount of AQP4 protein in the cerebellum compared with the cortex (*p* < .001), diencephalon (*p* < .001), hippocampus (*p* < .001), and the brainstem (*p* < .05). The brainstem demonstrated significantly higher AQP4 levels than both the cortex and hippocampus (*p* < .01), but not the diencephalon. No statistically significant difference between the cortex, diencephalons, and hippocampus was observed.

### AQP4 Expression in the Wild Type and Knockout Mouse Brain

**Figure 2.**
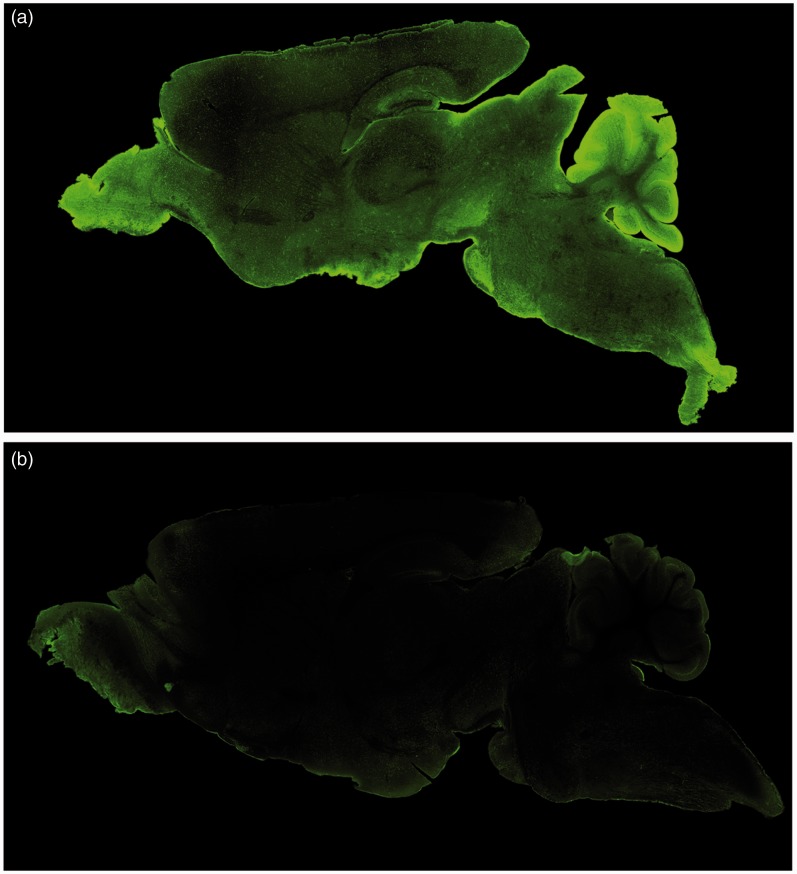
AQP4 immunoreactivity in the wild-type and AQP4 knockout mouse whole brain. 2× confocal images from sagittal brain slices were merged together to form a whole brain image of the mouse brain. (a) AQP4 immunoreactivity in a sagittal slice from a wild-type mouse brain. (b) AQP4 immunoreactivity in an AQP4 knockout mouse.

Consistent with the Western blot data, AQP4 immunoreactivity was high in various regions of the brainstem relative to the levels seen in the hippocampus and throughout the cortex and was highest in the cerebellum (Figure 2(a)). The hypothalamus and thalamus exhibited AQP4 labeling similar to that of the hippocampus and cortex. Most notably, AQP4 was uniformly distributed on astrocytic end-feet surrounding capillaries throughout the entire brain and any region associated with CSF including ependymal and glia limitans. Expression was homogenous throughout the cortex but exhibited heterogeneous sublayer-specific expression in other brain regions, such as the hippocampus and cerebellum. AQP4 knockout mice exhibited no AQP4 immunoreactivity (Figure 2(b)).

### AQP4 Expression in the Mouse Forebrain

**Figure 3.**
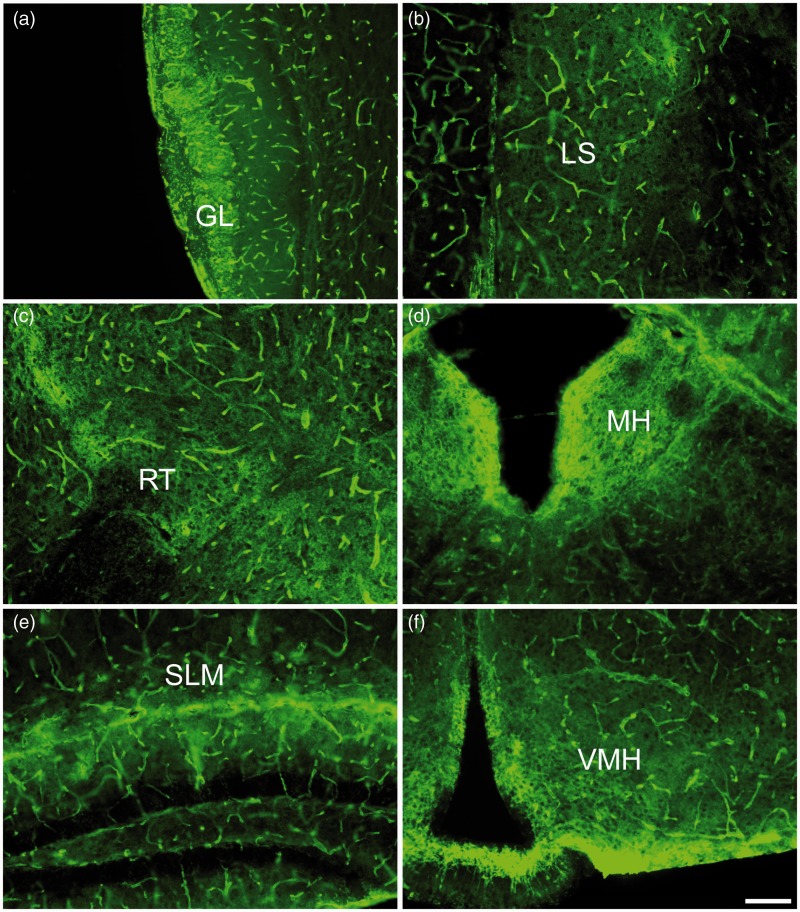
AQP4 immunoreactivity within forebrain regions, 10× images. (a) Glomerular layer of the olfactory bulb (GL). (b) Lateral septal nucleus (LS). (c) Reticular thalamic nucleus (RT). (d) Medial habenula (MH). (e) Stratum lacunosum moleculare (SLM) of the hippocampus. (f) Ventromedial hypothalamus (VMH). Scale bar: 100 µm.

In the most rostral part of the brain, the olfactory bulb, AQP4 immunoreactivity was richly expressed in the glomerular layer (Figure 3(a)). Both astrocytic processes and end-feet surrounding the capillaries within the glomerular layer densely expressed AQP4. Other layers of the olfactory bulb demonstrated much less parenchymal AQP4 immunoreactivity, although AQP4 was homogenously expressed around blood vessels throughout all layers of the olfactory bulb.

The medial olfactory area, the septal nuclei, receives several reciprocal connections from various brain regions including the olfactory bulb and the hippocampus. In the lateral septal nucleus, bush-like networks of AQP4 immunoreactivity were observed throughout, suggesting that astrocytic processes were well labeled with AQP4 (Figure 3(b)). Similarly, AQP4 staining was pronounced on the astrocyte processes within the thalamic nuclei, with rich AQP4 stain found in particular within the reticular thalamic nucleus (Figure 3(c)), a diencephalic GABAergic structure.

The habenula is a region of the brain that is reciprocally connected to the pineal gland and is thought to play a role in behavioral choices and responses to pain, stress, anxiety, sleep, and reward (Hikosaka, 2010). In the medial habenula (MH), by far, the most intense AQP4 immunoreactivity was observed around the ependymal cells lining the third ventricle with substantial immunoreactivity also seen in the adjacent glial cell processes within the habenular nucleus (Figure 3(d)). Intense staining outlined the cells in the MH suggesting cell membrane AQP4 expression.

The hippocampus exhibited laminar specific AQP4 immunoreactivity (Figure 3(e)) as previously observed (Hsu et al., 2011). In the CA1 region, AQP4 was intensely stained across the hippocampal fissure and within the stratum lacunosum moleculare (SLM). The dentate gyrus was largely absent of AQP4 staining with the exception of astrocytic end-feet and astrocytic processes that protruded into this neuronal layer. The CA1 stratum radiatum and hilus of the dentate gyrus both exhibited bush-like networks of AQP4 staining on astrocytes, although it was not as pronounced as that seen in the CA1 SLM. Of note, the staining of blood vessels throughout the hippocampus was not laminar-specific but, instead, was uniformly distributed throughout all layers.

Similar to what was observed in the MH, AQP4 staining was intense around the third ventricle and the glia limitans in the ventromedial hypothalamus (Figure 3(f)). Throughout the hypothalamus, a structure central to neuroendocrine function, AQP4 immunoreactivity was evident on astrocytic processes and end-feet surrounding capillaries. This staining, however, was not as prominent as the staining seen on glial cells lining the third ventricle.

### AQP4 Expression in the Mouse Brainstem

**Figure 4.**
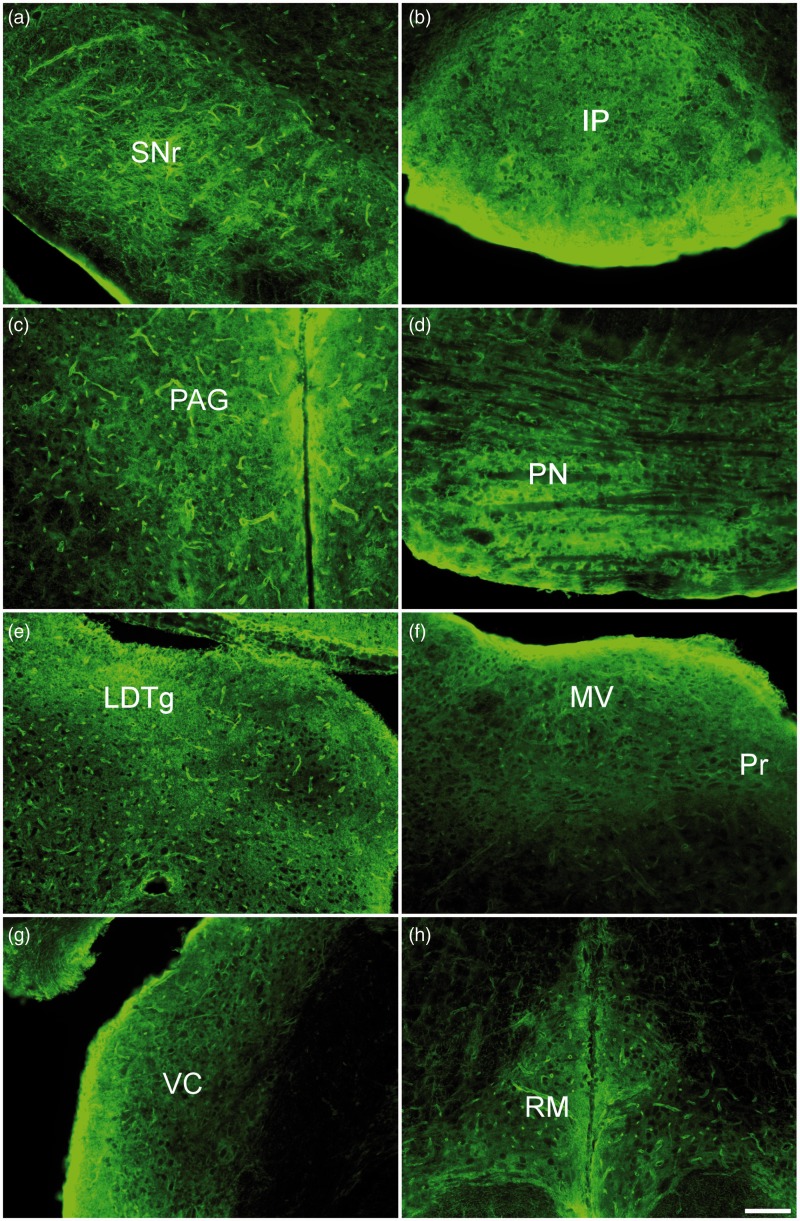
AQP4 immunoreactivity within the brainstem, 10× images. (a) Substantia nigra pars reticulata (SNr). (b) Interpeduncular nucleus (IP). (c) Periaqueductal gray (PAG). (d) Pons (PN). (e) Lateral dorsal tegmental nucleus (LDTg). (f) Medial vestibular nucleus (MV) and nucleus prepositus (Pr). (g) Ventral cochlear nucleus (VC). (h) Raphe magnus (RM). Scale bar: 100 µm.

Within the midbrain regions, AQP4 expression was prominent in cells surrounding ventricular and cisternal spaces (Figure 4). The substantia nigra of the basal ganglia exhibits robust AQP4 expression among the astrocytic end-feet surrounding the capillaries, astrocytic membranes, and the branched processes (Figure 4(a)). Of note, intense astrocytic AQP4 immunoreactivity is seen throughout the substantia nigra pars reticulata (SNr). In the substantia nigra pars compacta (SNc), AQP4 staining is not as intense, but it is still present on both the capillaries and the astrocytic membranes and processes. Interestingly, the substantia nigra is known to be involved in processes associated with movement, reward, addiction, and degeneration of the SNc is a hallmark of Parkinson’s disease.

Intense AQP4 immunoreactivity was found along the glial limiting membranes lining the caudal edge of the interpeduncular nucleus (Figure 4(b)), an area found at the base of the midbrain tegmentum that is associated with dopamine release and the regulation of rapid eye movement sleep. Within this region, homogenous AQP4 staining was found on astrocytes, with distinct branch-like processes seen throughout. Compared with other regions of the midbrain, fewer capillaries were observed in the interpeduncular nucleus, determined by the relatively low abundance of AQP4-positive astrocytic end-feet.

AQP4 labeling was densely along the cerebral aqueduct within the periaqueductal gray (Figure 4(c)), a brainstem region associated with pain modulation. Along the aqueduct, astrocytic membranes, processes and end-feet were all intensely labeled with AQP4. Lateral to that, AQP4-postive astrocytic bush-like processes and blood vessels were present; however, they were less prominent.

In the pons, intense AQP4 immunoreactivity was interspersed with patches of little to no AQP4 immunoreactivity (Figure 4(d)). This streak-like staining, in conjunction with the lack of capillaries, was unique to this region of the brain. The appearance suggests dark nonstained white matter pathways known to cross the pons and pockets of intense AQP4 immunoreactivity outlining cells (likely pontine reticular formation nuclei). This is in contrast to the robust staining seen in the lateral dorsal tegmental nucleus (Figure 4(e)), a part of the brain that, like the interpeduncular nucleus, is associated with the modulation of rapid eye movement sleep. In the lateral dorsal tegmental nucleus, capillaries were prominent and branch-like processes labeled with AQP4 were clearly present throughout.

Marked AQP4 staining in the hindbrain was seen along the ependymal surfaces lining the fourth ventricle in the medial vestibular nucleus and nucleus prepositus (Figure 4(f)), regions associated with eye movement and gaze holding, respectively. The glia limitans coating the lateral edge of the ventral cochlear nucleus (Figure 4(g)), closest to the flocculus, featured equally as prominent AQP4 immunoreactivity. Striking AQP4 staining was seen throughout the vestibular nucleus, but very little AQP4 stain was observed more medial to the ventral cochlear nucleus. Along the raphe magnus, another region associated with pain modulation, AQP4 is brightly stained (Figure 4(h)).

### Cerebellar Expression of AQP4

**Figure 5.**
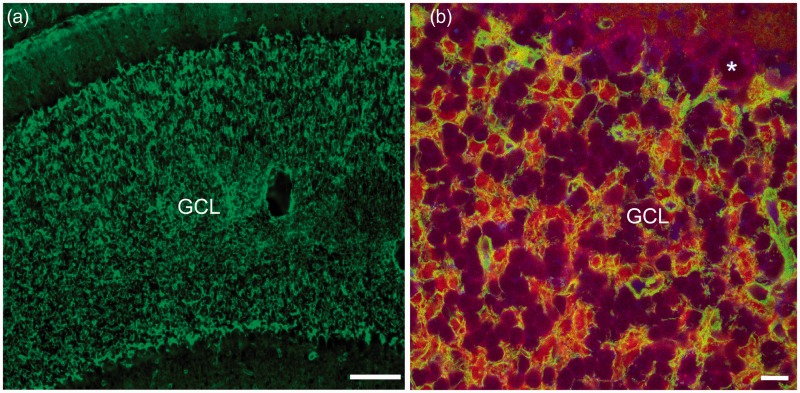
AQP4 and brevican immunoreactivity within the cerebellum. (a) Strong AQP4 signal is observed in the cerebellar granule cell layer (GCL; 10×). Scale bar: 100 µm. (b) Higher-power image of AQP4 (green), brevican (red), and Nissl (blue) labeling in the cerebellum (63×). Example of a Purkinje cell denoted with an asterisk (*). Scale bar: 10 µm.

The unique architecture of the cerebellum makes AQP4 immunoreactivity look distinct from any other brain region (Figure 5(a)). AQP4 immunoreactivity was rich in the granule cell layer and prominent surrounding the blood vessels within the cerebellum. The Purkinje cell layer was nearly devoid of AQP4 staining except for some astrocytic processes extending from the granule cell layer. The molecular layer had abundant astrocytic AQP4 staining. In both the granule cell and molecular layers, AQP4 was seen on astrocyte cell membranes and their branched processes.

Brevican is a chondroitin sulfate proteoglycan that has previously been shown to be synthesized by and expressed on cerebellar astrocytes (Yamada et al., 1997). Confocal triple-labeled immunohistochemistry for AQP4 (green), brevican (red), and Nissl (blue) clearly demonstrated colocalization of AQP4 and brevican on cerebellar astrocytes (Figure 5(b)). Again, AQP4 was absent from Purkinje cells but was abundant throughout the granule cell layer.

## Discussion

In this study, we used Western blotting and immunohistochemistry to elucidate the expression pattern of AQP4 throughout the mouse brain. Prior studies have demonstrated AQP4 expression in various regions of the brain (Nielsen et al., 1997; Rash et al., 1998; Wen et al., 1999; Frigeri et al., 2001; Nagy et al., 2002; Amiry-Moghaddam et al., 2003, 2004a; Costa et al., 2007; Hsu et al., 2011) and spinal cord (Rash et al., 1998; Oshio et al., 2004; Vitellaro-Zuccarello et al., 2005); however, this is the first comprehensive neuroanatomical study of AQP4 localization and protein levels in the rodent brain. We also demonstrate marked expression and colocalization of AQP4 with brevican, a proteoglycan found on the surface of astrocytes in the cerebellum.

The expression pattern of AQP4 suggests a specialized role in mediating the water movement between glial cells and cavities containing CSF and the intravascular space. We, like others, have found highly polarized AQP4 expression, with intense immunoreactivity on glia limitans boarding the subarachnoid space and ventricles, subpopulations of ependymocytes, pia, and perivascular glial end-feet surrounding capillaries (Nielsen et al., 1997; Amiry-Moghaddam et al., 2003; Goren et al., 2006). Although AQP4 has been found on ependymal cells (Badaut et al., 2000b), it was completely absent from tanycytes, a class of ependymal cells found in the floor of the third ventricle that contact hypothalamic neurons and blood vessels (Badaut et al., 2002). AQP4 knockout mice lack the ability to properly maintain water homeostasis and, therefore, are more prone to neurological decline in vasogenic edema (Papadopoulos et al., 2004).

In previous studies, AQP4 immunoreactivity has been found close to blood vessels and on astrocyte processes in the corpus callosum (Badaut et al., 2000b, 2002, 2014). AQP4, like AQP9, has also been found on other white matter tracts such as the anterior commissure and optic chiasm (Badaut et al., 2002). AQP9 exhibited a similar distribution to AQP4 and is thought to play a role in aiding AQP4 function. Both AQP4 and AQP9 have been found on astrocytic processes in periventricular regions of parenchyma and in glia limitans bordering subarachnoid space (Badaut et al., 2002). More recently, however, studies of AQP9 knockout mice revealed that in addition to astrocytes and ependymal cells, AQP9 is also expressed on catecholaminergic neurons (Mylonakou et al., 2009; Badaut et al., 2014).

We have demonstrated a region-specific expression pattern of AQP4. Robust staining was found on astrocytic membranes and processes in the lateral septal nuclei, reticular thalamic nucleus, hippocampal fissure, and SLM layer of CA1, SNr, interpeduncular nucleus and throughout the granule cell layer of the cerebellum. Very little AQP4 immunoreactivity was found medial to the ventral cochlear nucleus and in predominantly neuronal areas, such as the dentate granule cell layer of the hippocampus and the Purkinje cell layer of the cerebellum.

The heterogeneous pattern of AQP4 expression throughout the brain suggests various functional roles for AQP4 in addition to its role in water movement across cell membranes. For example, we observed robust AQP4 staining in the ventral cochlear nuclei. Previously, AQP4 knockout mice were found to have impaired hearing (Li and Verkman, 2001; Mhatre et al., 2002), which was interpreted as suggesting a role for AQP4 in ion and water homeostasis in the inner ear. However, our findings suggest that deficiency of AQP4 in the ventral cochlear nuclei or central auditory pathways could also play a role. Similarly, in the olfactory bulb, AQP4 expression was highest in the glomerular layer, the characteristic multicellular synaptic unit of the olfactory bulb, which agrees with previous findings (Sorbo et al., 2007). Interestingly, AQP4 knockout mice exhibit impaired olfaction (Lu et al., 2008), which was interpreted as a deficit in the olfactory epithelium, but our results suggest that deficiency of AQP4 in the glomerular layer of the olfactory bulb may be responsible for this phenotype. The septal nucleus is connected to both the olfactory bulb and the hippocampus, a structure associated with learning and memory formation. Here, we have shown abundant AQP4 expression in the septal nuclei, hippocampus, and glomerular layer of the olfactory bulb. A correlation between AQP4 and olfactory learning has not been shown but would be interesting to explore. It is of interest that AQP4 expression is particularly high in the thalamic reticular nucleus, a structure hypothesized to be important in selective attention and other functions related to consciousness (Crick, 1984; Pinault, 2004).

The hippocampus is a structure critical to cognitive function and long-term memory formation. Our current findings agree with previous results suggesting laminar-specific expression of AQP4 in the hippocampus, with highest levels of AQP4 staining near the hippocampal fissure and in the SLM (Costa et al., 2007; Hsu et al., 2011). Previous studies have shown a role for AQP4 in synaptic plasticity. Specifically, AQP4-deficient mice exhibit impaired long-term potentiation and long-term depression without any change in basal transmission (Skucas et al., 2011; Scharfman and Binder, 2013). Furthermore, marked AQP4 downregulation has been observed in an animal model of temporal lobe epilepsy (Lee et al., 2012), and AQP4-deficient mice have slowed K^+^ kinetics and increased seizure duration (Binder et al., 2006). It is well-known that patients with temporal lobe epilepsy often exhibit cognitive deficits, particularly in hippocampal-dependent tasks such as spatial memory (Bell et al., 2011; Amlerova et al., 2013; Brooks-Kayal et al., 2013; Chin and Scharfman, 2013). These data, together with recent findings that AQP4 modulates extracellular [K^+^] during synaptic stimulation in the hippocampus (Haj-Yasein et al., 2014), suggest that AQP4 is essential for synaptic plasticity.

The hypothalamic magnocellular neurosecretory system has been implicated in both neuronal and glial plasticity (Hatton, 1986). A previous study has demonstrated that AQP4 staining is abundant in the rat magnocellular hypothalamic nuclei (Badaut et al., 2000a). In addition, high AQP4 mRNA levels have been observed in thalamic and hypothalamic regions (Venero et al., 1999). Future studies should examine AQP4 expression and regulation in subregions of the hypothalamus that have distinct roles in neuroendocrine regulation and plasticity. The polysialylated embryonic form of neural cell adhesion molecule (PSA-NCAM) has been shown to be required for the induction of synaptic plasticity (Muller et al., 1996). Therefore, it would be of interest to examine the distribution of PSA-NCAM in hypothalamic subregions and its colocalization with AQP4. Furthermore, it would be interesting to examine hypothalamic synaptic plasticity in AQP4 knockout mice, as has been done in the hippocampus (Skucas et al., 2011).

The periaqueductal gray plays a major role in pain and analgesia. We found abundant levels of AQP4 expressed throughout this region. Mice lacking AQP4 have increased pain thresholds to thermal and chemical stimulation, but not mechanical stimulation (Bao et al., 2010). Further supporting a role for AQP4 in pain modulation, Nesic et al. (2005) found increased levels of AQP4 mRNA and protein in rats exhibiting central neuropathic pain. It would be interesting for future studies to further explore the role of AQP4 in brain regions associated with pain modulation, such as the periaqueductal gray or the raphe magnus, in which AQP4 immunoreactivity is also prominent.

Similar to AQP4, AQP1 has also been implicated in pain perception. AQP1 knockout mice exhibited reduced thermal, inflammatory, chemical, and cold pain perception but did not differ in response to mechanical stimuli (Oshio et al., 2006; Zhang and Verkman, 2010). Although AQP1 has been considered the major water transport protein of the choroid plexus and is thought to play a role in the secretion of CSF (Bondy et al., 1993; Nielsen et al., 1993; Hasegawa et al., 1994; Badaut et al., 2002; Oshio et al., 2003; Longatti et al., 2004; Oshio et al., 2005; Fukuda et al., 2012), it was also been found on dorsal root ganglion (DRG) neurons (Zhang and Verkman, 2010, 2015). Recent studies have implicated AQP1 in DRG axonal growth and regeneration as well as osmotic water permeability in isolated DRG neurons (Zhang and Verkman, 2010, 2015).

Several anatomical regions of the brain are associated with reward and addiction pathways, including the ventral tegmental area, medial prefrontal cortex, hippocampus, ventral striatum (including the nucleus accumbens), and the amygdala (Luthi and Luscher, 2014). Projections connecting these various brain regions are dopaminergic in nature. Previous studies have shown that dopamine can decrease AQP4 water permeability (Zelenina et al., 2002) and AQP4 protein expression (Kuppers et al., 2008). It has also been shown that AQP4 deficiency can increase K^+^-stimulated release of striatal dopamine (Ding et al., 2007). Recent studies have shown that AQP4-deficient mice have attenuated morphine tolerance, inhibited development of morphine physical dependence and impaired morphine analgesia (Wu et al., 2008; Chen et al., 2010). In addition, chronic treatment with morphine decreased spinal AQP4 expression in a rodent model (Chen et al., 2010). An interesting study examining cocaine-induced locomotion found that AQP4 knockout mice exhibited attenuated locomotor activity in response to cocaine stimulation (Li et al., 2006). Of note, whereas the ventral tegmental area is associated with reward and addiction, the substantia nigra of the midbrain, which exhibits robust AQP4 immunoreactivity and utilizes dopamine, is associated with movement as well as reward and addiction. These studies, taken together, suggest the involvement of AQP4 in addictive behavior.

Of all brain regions, AQP4 was most abundantly expressed in the cerebellum, which is associated with motor control and motor learning (Therrien and Bastian, 2015). We have shown intense AQP4 immunoreactivity throughout the cerebellum; however, very little is known about the relationship between cerebellar AQP4 and motor control. Given the findings in the hippocampus that AQP4-deficient mice exhibit impaired synaptic plasticity (Skucas et al., 2011; Scharfman and Binder, 2013), it would be interesting to analyze the physiology of cerebellar slices from AQP4-deficient mice for alterations in cerebellar synaptic plasticity (e.g., cerebellar long-term depression), and more generally to assess AQP4-deficient mice for deficits in cerebellar learning paradigms.

Brevican, an extracellular matrix proteoglycan, is synthesized by astrocytes and retained on their surface throughout the adult rodent brain, but its highest level of expression and spatial organization is within the cerebellar cortex (Yamada et al., 1997). Brevican has been shown to parallel the gliotic response in central nervous system injury, suggesting a role of brevican in reactive gliosis (Jaworski et al., 1999; Thon et al., 2000). We demonstrate colocalization of brevican and AQP4 in the cerebellum. Together with other studies demonstrating colocalization of AQP4 and astrocyte markers (GFAP, S-100β; Hsu et al., 2011), our current data clearly indicate cell-type specificity of AQP4 expression in astrocytes.

It is well-known that AQP4 is tethered to the membrane by α-syntrophin, a component of the dystrophin protein complex. Mice deficient in α-syntrophin exhibited reduced AQP4 expression on perivascular astrocytic end-feet membranes and increased levels on membranes facing the neuropil (Neely et al., 2001; Amiry-Moghaddam et al., 2004b). Dystrophin-independent pools of AQP4 have been found in the granular cell layer of the cerebellum, the subpial end-foot layer, and in ependymal cells (Nicchia et al., 2008). In addition, AQP4 and α-syntrophin frequently colocalized at astrocytic membranes, particularly at perivascular astrocyte end-foot processes, suggesting a linkage between these two molecules (Inoue et al., 2002; Masaki et al., 2010). Complimentary to these findings, deletion of the dystroglycan gene resulted in a reduction of AQP4 perivascular expression and a loss of the formation of orthogonal arrays of particles (OAPs; Noell et al., 2011). The proteoglycan agrin has been implicated in the polarized distribution of AQP4, specifically restricting OAPs to end-feet membranes (Warth et al., 2004; Noell et al., 2007, 2009). Studies of agrin knockout mice have demonstrated that AQP4 expression was not altered, but the formation of OAPs was decreased (Noell et al., 2009). More recently, a study involving endothelial-astrocyte cocultures demonstrated that application of agrin gave rise to a pronounced polarization and membrane compartmentalization of AQP4 (Camassa et al., 2015). Taken together, these data suggest that dystrophin and agrin proteins may be responsible for the clustering of AQP4 around blood vessels.

In summary, AQP4 was densely expressed on astrocyte processes surrounding blood vessels (astrocyte end-feet) and in regions of the brain associated with CSF flow, including ventricles, subarachnoid space, and ependymal cells. Regionally, AQP4 protein expression was highest in the cerebellum, where it was strongly expressed on the dense astrocyte network throughout the granule cell layer. Throughout the entire brain, AQP4 not only demonstrated region specificity but also laminar specificity within individual structures, further supporting the developing concept of astrocyte heterogeneity (Matyash and Kettenmann, 2010; Hoft et al., 2014; Bayraktar et al., 2015). Reduced levels of AQP4 are associated with a number of functional defects, such as impaired hearing and olfaction, and neurological disorders, including edema, epilepsy, and stroke. Thus, investigation of the local functional relationship between AQP4 expression and specific anatomical regions and circuits could offer new insight into the diverse roles of AQP4 in the brain.

## Summary

Aquaporin-4 is a glial water channel that is responsible for water fluxes and ion homeostasis in the brain. Here, we characterized the distribution of aquaporin-4 and found that expression was high in the cerebellum and areas associated with water movement.
